# Supplementary material for: Tele-medicine controlled hospital at home is associated with better outcomes than hospital stay
Source: PLoS One. 2024 Aug 19;19(8):e0309077. doi: 10.1371/journal.pone.0309077 (PMC11332917; doi:10.1371/journal.pone.0309077)
Supplement: Attachment — Submitted filename: Response to Reviewers.docx [file pone.0309077.s001.docx]

**Response to Reviewers**

Dear editor. It is an honor for us to submit our revised manuscript with responses to the reviewers’ comments. We see these as improving the quality of our manuscript and we thank you for considering our work for publication.

On behalf of all authors,

Prof. Gad Segal, MD

| Comment # | Reviewer comment | Authors answer | Place in R1 |
| --- | --- | --- | --- |
| **Academic editor** | | | |
| 1 | I believe that it is very important to carry out a statistical analysis and cost analysis. | Indeed, these comments were also addressed as a result of the reviewers’ comments. | Lines 356 – 364. |
| 2 | I also suggest evaluating the effect of Artificial Intelligence on telehealth. | Indeed, these comments were also addressed as a result of the reviewers’ comments. | Lines 365 – 381. |
| 3 | Moreover, I suggest improving the bibliography. | The bibliography was expanded in accord with the reviewers’ comments. | Lines 407 – 547. |
| **Reviewer 1** | | | |
| General comment | The article is interesting and well written. To improve its quality, I have some suggestions: | | |
| 1 | authors should highlight how results can change depending on the type of technology, mode of telemedicine session administration and healthcare professional involved | Insights relating to this point were added to the discussion. | Lines 261 – 265. |
| 2 | authors should perform an inferential analysis with the use of regression techniques to investigate how outcomes can be associated to baseline characteristics and socio-economic indicators (i.e. income, education, marital status...) | As a retrospective study, not all data were available for analysis and comparison between groups. Relating specifically to socio-economic patients’ characteristics, we know that all patients came from a similar background: this is both an advantage relating to the between-groups’ comparison and a disadvantage when relating to the generalizability of our results. All the above were added to the “discussion” and mentioned in the “limitations” paragraphs. | Lines 356 – 364 + lines 395 – 397. |
| 3 | authors should perform scenario analysis or at least discuss on whether results are sensitive and generalizable to other jurisdictions | Indeed, we added and designated paragraph to the discussion. | Lines 335 – 355. |
| 4 | authors should include a cost analysis highlighting how costs and in general resource use can change depending how the program is dimensioned (i.e.: initial investment requires fixed costs that should be compensated over different years, thus allowing to achieve a break even point) | As a retrospective study, it was not powered to produce a thorough cost-analysis. We did provide the relevant costs in the results chapter. | Lines 356 – 364. |
| **Reviewer 2** | | | |
| 1 | Dear Authors, thank you for your work. please add more references (eg: 10.3390/s23125408, 10.3390/ijerph181910328, 10.1093/eurpub/ckab165.159), they should be >50. | References were added. | Lines 67 – 71. |
| 2 | Did you consider AI effect on tele-health? | Indeed, this is an important component of or future and accordingly, we added a dedicated chapter to our discussion. | Lines 365 – 381. |
| **General / technical comments** | | | |
| We note that the grant information you provided in the ‘Funding Information’ and ‘Financial Disclosure’ sections do not match. When you resubmit, please ensure that you provide the correct grant numbers for the awards you received for your study in the ‘Funding Information’ section. | | We thank you for your attention. Correct grant information was provided upon re-submission.  Lines 401 – 402. | |
| Thank you for stating the following financial disclosure:  "The Research was supported in part by an Israel Science Foundation [Grant 277/21] and the Israel National Institute for Health Policy Research [Grant 2021/160/R]. Guarantor: No guarantees were given regarding this study."  Please state what role the funders took in the study.  If the funders had no role, please state: ""The funders had no role in study design, data collection and analysis, decision to publish, or preparation of the manuscript.""  If this statement is not correct you must amend it as needed.  Please include this amended Role of Funder statement in your cover letter; we will change the online submission form on your behalf. | | We thank you for your attention. The statement relating to the funders’ role was added.  Lines 402 – 44. | |
| We note that you have indicated that there are restrictions to data sharing for this study. For studies involving human research participant data or other sensitive data, we encourage authors to share de-identified or anonymized data. However, when data cannot be publicly shared for ethical reasons, we allow authors to make their data sets available upon request. For information on unacceptable data access restrictions, please see http://journals.plos.org/plosone/s/data-availability#loc-unacceptable-data-access-restrictions. | | We thank you for your attention. The de-identified data will become available upon request under acceptable data restrictions and on the terms and conditions made by the local IRB. | |
| If there are ethical or legal restrictions on sharing a de-identified data set, please explain them in detail (e.g., data contain potentially identifying or sensitive patient information, data are owned by a third-party organization, etc.) and who has imposed them (e.g., a Research Ethics Committee or Institutional Review Board, etc.). Please also provide contact information for a data access committee, ethics committee, or other institutional body to which data requests may be sent. | |  |  |
| If there are no restrictions, please upload the minimal anonymized data set necessary to replicate your study findings to a stable, public repository and provide us with the relevant URLs, DOIs, or accession numbers. Please see http://www.bmj.com/content/340/bmj.c181.long for guidelines on how to de-identify and prepare clinical data for publication. For a list of recommended repositories, please see https://journals.plos.org/plosone/s/recommended-repositories. You also have the option of uploading the data as Supporting Information files, but we would recommend depositing data directly to a data repository if possible. Please update your Data Availability statement in the submission form accordingly. | |  |  |
| While revising your submission, please upload your figure files to the Preflight Analysis and Conversion Engine (PACE) digital diagnostic tool, <https://pacev2.apexcovantage.com/>. PACE helps ensure that figures meet PLOS requirements. To use PACE, you must first register as a user. Registration is free. Then, login and navigate to the UPLOAD tab, where you will find detailed instructions on how to use the tool. If you encounter any issues or have any questions when using PACE, please email PLOS at <a href="mailto:figures@plos.org">figures@plos.org. Please note that Supporting Information files do not need this step. | | Figures were uploaded as requested. | |
